# Nursing students’ experiences of clinical assessment at a university in South Africa

**DOI:** 10.4102/hsag.v28i0.2161

**Published:** 2023-02-03

**Authors:** Gabieba Donough

**Affiliations:** 1 School of Nursing, Faculty of Community and Health Sciences, University of the Western Cape, Cape Town, South Africa

**Keywords:** clinical accompaniment, clinical assessment, clinical supervision, clinical supervisor, undergraduate nursing students

## Abstract

**Background:**

Nursing education includes both classroom and clinical teaching. The clinical teaching was explored through this research. The successful training of the undergraduate nursing students can be attributed to effective clinical teaching and supervision and is determined by both training requirements and services provided. Although there have been several researches on clinical supervision, there is still a dearth of information of the realities of supervision regarding assessment of undergraduate nursing students. The authors’ original thesis formed the foundation of this manuscript.

**Aim:**

This study aimed to explore and describe nursing students at the undergraduate level experiences regarding clinical supervision.

**Setting:**

The research was conducted at a nursing school at a South African university.

**Method:**

After ethical clearance, focus group interviews were conducted to explore undergraduate nursing students’ experiences of clinical supervision using a descriptive design and qualitative approach. Two qualified practitioners in the field collected the data. A purposive method was utilised to select nine participants from each year’s level of education. Enrolled undergraduate nursing students at the institution under study formed the inclusion criteria. Utilising content analysis, the interviews were analysed.

**Results:**

The findings confirmed the students’ experiences of clinical supervision and voicing their concerns regarding clinical assessment versus a developmental training; clinical teaching, learning and assessment and formative assessment procedures.

**Conclusion:**

A responsive clinical supervision system to strategically respond to the needs of undergraduate nursing students will aid in developmental training and assessment.

**Contribution:**

Understanding of the realities of clinical teaching and supervision regarding clinical assessment and development of undergraduate nursing students.

## Introduction

Clinical assessment serves to groom and equip nursing students to function as responsible, ethical and safe nurse practitioners (Wu et al. 2018). Clinical assessment is conducted in the form of formative and summative assessment. Exposure to clinical practice can strengthen their clinical competencies (Motsaanaka, Makhene & Ally 2020). Clinical supervisors facilitate student learning at both learning institution and clinical placement (South African Nursing Council 2013).

In South Africa, the provision of quality undergraduate clinical supervision is connected to the clinical supervisor’s function in the health profession (Voges & Frantz 2019). The clinical supervisor has various functions that include being an assessor, educator, mentor and clinical supervisor (Bearman et al. 2018; Donough, Van der Heever & Stellenberg 2014; Voges & Frantz 2019). In nursing, these functions are performed by registered nurses, nurse educators and clinical preceptors (Thurling, Muthathi & Armstrong 2017). They are also referred to as facilitators, mentors and teachers that do accompaniment (South African Nursing Council 2013; Solheim, Plathe & Eide 2017). Clinical accompaniment is a planned process enabling clinical supervisors to provide students with direct support and assistance in a clinical setting, ensuring that learning objectives are achieved (South African Nursing Council 2013). The clinical supervisor assesses the student to understand their performances and determine their level of competence (South African Nursing Council 2013; Wu et al. 2015). Competence can be described as the action required to fulfil the nursing role (Fukada 2018).

Undergraduate nursing students train to become qualified professional nurses, who play a crucial role in providing healthcare services and are frequently the only source of help in many regions (Bell & Brysiewicz 2020). When they train at a clinical placement, nursing students are under the guidance of the clinical supervisor (Collier 2018; South African Nursing Council 2013). Students are the recipients of clinical supervision, and hence their experiences can provide insight into the realities of clinical supervision regarding clinical assessment. In addition, Bearman et al. (2018) conducted a study to determine the aspects of clinical supervisors’ teaching practice that needs development. Findings revealed that clinical supervisors have challenges in managing clinical supervision functions with teaching responsibilities because of lack of time. The study further revealed that the clinical supervisors were lacking in clinical assessment skills. Therefore, this manuscript focuses on nursing students’ experiences of clinical assessment from the student’s perspective.

## Purpose of the study

The study’s purpose was to explore and describe the experiences of undergraduate nursing students on clinical supervision.

## Research method and design

Using a descriptive design and a qualitative approach, the experiences of these undergraduate students were explored. This approach was deemed appropriate as it allowed for the exploration of many individual experiences, including viewpoints, problems, learning and the significance of the experiences for each individual. Focus group interviews were used to collect data (Creswell 2014).

### Research setting

The study was carried out at a School of Nursing in South Africa. This School of Nursing is situated in a higher education institution’s (HEI’s) Faculty of Community and Health Sciences, which provides undergraduate and postgraduate programmes. The Baccalaureus of Nursing degree in accordance with Regulation No. 425 (R425), on which this study was based, was part of the undergraduate curriculum on offer (SANC 2021). Clinical supervisors are employed by the institution with the responsibility to conduct clinical teaching and accompaniment.

### Population and sampling

A purposive method of sampling was used. Using this technique, the researcher was able to select the students who had relevant experiences to the topic being researched (Etikan, Musa & Alkassim 2016). At the time of the study, the population included *N* = 1001 undergraduate nursing students registered in the 4-year undergraduate programme at the selected School of Nursing (Donough et al. 2014). The coordinators at the institution under study helped with the selection of the undergraduate nursing students who met the inclusion criteria. This was performed after ethical clearance when the Health Research Ethical Committee at Stellenbosch University and the head of the department at institution under study gave approval.

The focus groups included nine registered undergraduate students per year level of the Bachelor’s degree in Nursing as part of the inclusion criteria. Sequentially, there were nine first-year students, nine second-year students, nine third-year students and nine fourth-year students who took part in the group interviews. Furthermore, the inclusion criteria required that the students to have worked at a clinical placement setting where they received clinical supervision. Thirty-six students from nursing formed the sample. According to Creswell and Poth (2016), in qualitative research, the number of participants is sufficient once information in the particular subject area has reached saturation and had been verified. In addition, instead of the number of participants, information that is of quality is the main focus of qualitative research.

### Data collection

A semi-structured interview guide was used, which allowed for different replies to predetermined questions and provided researchers with elaborate answers (Creswell 2014). The interview guide included open-ended questions about the students’ experiences with clinical supervision. The study’s purpose served as the foundation for the questions and probing words. The participants agreed in writing for the interviews to be recorded.

To prevent the risk of conflict, data were gathered by two practitioners in the field who were not connected to the identified institution, instead of the researcher who at the time worked at the institution. This assured trustworthiness. Both practitioners in the field were skilled interviewers and held master’s degrees in nursing. This gave the participants the chance to express their opinions without worrying about the researcher’s potential influence.

During the interview, the type of questions posed were open ended, for example, to share their experience regarding clinical supervision. Fairness, consistency across supervisors, clinical demonstrations and assessments were the words used for probing.

The interviews took place at a time and place that were acceptable for the participants. The participants preferred the institution’s premises and deemed it as convenient. To guarantee that every participant understood the questions, interviews were carried out in English. The practitioners in the field made notes on important actions seen during the interviews in addition to recording information with a recording device. Data saturation was reached when information redundancy occurred, and hence no additional interviews were conducted (Braun & Clarke 2021).

### Data analysis

The recorded interviews of the four focus groups were verbatim transcribed to increase the trustworthiness. Utilising Blanche et al.’s (2006) five-step approach for content analysis: in Step 1 the transcriptions were repeatedly read and the recordings listened to fully understand the experiences and become familiar with the information known as familiarisation and immersion. The information gathered from the interviews was dissected, scrutinised and reviewed in Step 2 to identify trends, similarities and discrepancies known as inducing themes. The theme that inevitably accompanied the data were organised into a theme and subthemes. By determining the key messages in each transcript and labelling those sections of text, Step 3 of the transcription process was accomplished. The author was able to investigate the theme in further detail and make changes to the coding system in Step 4 through elaboration. The theme was re-evaluated in Step 5 for potential misunderstandings, whether significant concerns were missed and whether the author’s prejudices may have affected the final theme, known as interpretation and checking. The method of inductive reasoning involves starting with particular facts and gradually move to general based on what has been observed (Woo, O’boyle & Spector 2017). In the light of this, the researcher was able to gather facts by studying the events and then draw inferences based on this data, as stated by Woo et al. (2017).

### Measures of trustworthiness

Trustworthiness approach, by using the accurateness and integrity of the findings, outlined by Lincoln and Guba (1988), which includes credibility, transferability, conformability and dependability: In order to establish credibility through member checking, participants were contacted and given the chance to assess the transcripts’ content and discuss the main themes and subthemes as well as their interpretation. Transferability was achieved by giving in-depth explanations of the sample and data collecting and analysis methods, how the information was acquired, the author’s relationship with the participants and the rationale for the use of practitioners in the field in the data collection. Conformability was achieved by ensuring that the findings accurately reflect the opinions of the participants and that the data appropriately reflect the information they gave. The direct quotations taken from the transcripts suggest confirmability. Dependability by using the transcripts as a reference, the author confirmed the veracity of the interview recordings made by the field researchers. In addition, an expert audio transcriptionist completed the transcription of the audio recordings and proofread all the documents.

### Ethical considerations

The Health Research Ethical Committee at Stellenbosch University (Ethics reference no.: S12/05/132) gave clearance for the study’s ethical conduct. The head of the department at the institution under study gave permission to conduct the study at the institution as well as permission to use the students as participants. To maintain their anonymity, neither the name of the students nor the institution was publicly disclosed. Participants were given alias names such as ‘participant 1’ throughout the interview. In addition, the transcripts of the interviews were managed using these codes. The recordings and transcripts that made up the electronic versions of the material are code encrypted. The data files are deleted after 5 years.

## Findings and discussion

One main theme and three subthemes emerged from the interviews related to the nursing students’ experiences of clinical assessment in the undergraduate nursing programme at a School of Nursing in South Africa (see Table 1).

**TABLE 1:** Theme and sub-themes.

Theme	Sub-themes
1. Clinical assessment by the clinical supervisor	1.1: Clinical assessment versus a developmental training 1.2: Clinical teaching, learning and assessment 1.3: Formative assessment procedures

### Theme 1: Clinical assessment by the clinical supervisor

The purpose of this study was to explore how undergraduate nursing students experienced clinical supervision. The level of responses signified the clinical supervisor’s assessments. This theme refers to the clinical assessments of the participants conducted by the clinical supervisors. Clinical assessment or making a judgement on students’ performance is important and promotes learning (Bearman et al. 2018). The findings regarding clinical assessment by the clinical supervisor are grouped into three following subthemes.

#### Sub-theme 1.1: Clinical assessment versus a developmental training

Clinical supervisors play a key role in helping students integrate the knowledge of theory and practice (Voges & Frantz 2019). To accomplish this, clinical supervisors should spend time accompanying students in the clinical setting and guide students’ learning through developmental training and assessment (Tuomikoski et al. 2020). Development training refers to training designed to advance knowledge, skill and attitude (Jayasekara et al. 2018). Assessment forms part of learning (Pellegrino 2018). The participants, however, expressed their concern about their limited time spend with clinical supervisors as evidenced by the following citation:

‘… we know some of them [*supervisors*] are not going to pitch.’ (Group 3, Participant 6, Focus group interview)

‘… from last semester… there’s no one [*supervisor*] came to see us and how we doing.’ (Group 1, Participant 6, Focus group interview).

The responses gave the impression that certain supervisors were not always available for accompaniment and to guide students. It raised concerns regarding the students’ access to developmental training and asking clarity-seeking questions from supervisors. Conversely, the South African Nursing Council establishes and upholds baccalaureate nursing standards and mandates that each student be supervised for at minimum an hour every 2 weeks (South African Nursing Council 1985, 2013). Furthermore, other participants expressed concerns about the shortened accompaniment sessions, indicating it was too brief and insufficient. Participants expressed a desire for additional bedside training:

‘Like some will just go and five minutes and they’ll be gone. So, I felt maybe they could have supported you in what actually happening on the ward.’ (Group 4, Participant 1, Focus group interview)

‘They don’t spend enough time with us so that we can have to ask them questions.’ (Group 3, Participant 1, Focus group interview)

Participants voiced concern regarding clinical supervisors that only conduct assessment processes rather than provide developmental training. The participants had the sense that clinical supervisors are assessment-focused evidenced by the following response:

‘I wish they would like not only come when we have an assessment to do… they only pitch when there’s something that you have to do… the only things I make sure I know is the vital signs, the wound care and that’s it. But the other stuff it’s like they’re not important because we’re not frequently showed how to do them, so I wish they would come when other days.’ (Group 1, Participant 2, Focus group interview)

The given response not only generated the sense that clinical supervisors were assessment focused but it also indicated that participants are aware of their need for developmental training. The participants in the different groups all concurred that actual clinical accompaniment sessions were necessary and some of the participants pointed out the limited accompaniment in this regard.

Identifying the absence of clinical supervisors could be the reason why participants receive little developmental training and only engaged clinical supervisors at assessments. Clinical supervisors should spend more time accompanying students in clinical settings and guide students’ learning through developmental training and assessment. Setting boundaries according to standards and guidelines can ensure that the accompaniment principles are applied. This might offer an answer to some of the issues experienced by undergraduate nursing students. This prompted the next subtheme, which is related to one of the functions of the clinical supervisor.

#### Sub-theme 1.2: Clinical teaching, learning and assessment

The function of the clinical supervisor is to demonstrate a procedure to the students as part of clinical teaching. The skills laboratory methodology enables clinical learning and teaching through demonstration, followed by practice and finally, an assessment of the student by the clinical supervisor (Kleinsmith 2017). The following comment relates to the process after demonstration and practice where the student must engage in self-directed learning before the clinical supervisor assesses:

‘… you need you to know your work, I need you to go and do the homework so that when you come back you are competent.’ (Group 2, Participant 7, Focus group interview)

Supporting students’ learning processes and developing them into self-directed learners requires effective clinical teaching (Tuomikoski et al. 2020). The participants, however, voiced that they did not receive much of development and guidance:

‘So basically, supervision needs a proper, a someone who is going to guide you through the process which is what I believe personally that in most, I would say forty percent of our supervisors lack that.’ (Group 3, Participant 2, Focus group interview)

Participants were able to articulate what they desired and expressed concern about the clinical supervisors’ lack of expertise. Participants further explained inconsistent clinical teaching and expressed their concerns:

‘… every supervisor has her way of doing things, of teaching us, so every time when you get a new supervisor you have to adapt on how maybe she wants … to do certain things, certain way.’ (Group 1, Participant 3, Focus group interview)

‘So, the one supervisor in the lab they will show you, this is the procedure, this is the way… but when you’re in the clinical facility, when I did my demonstration when I practice it, the supervisor was saying ‘no, you’re doing it wrong.’ (Group 4, Participant 1, Focus group interview)

The findings confirm the participants’ experiences of disparities of clinical procedures by different supervisors. Regardless of a practical workbook that includes instructions for carrying out clinical procedures, participants reported that supervisors would demonstrate the same procedure in different ways. The participants were affected by the supervisors’ inconsistencies and their different ways of doing a procedure:

‘… supervisors not being congruent whether demonstrations that they do so it’s different so that will actually mean that you’re marked down when you do your assessment.’ (Group 4, Participant 4, Focus group interview)

‘… one supervisor assesses this way and the other one assesses this way.’ (Group 3, Participant 2, Focus group interview)

The given comments raise questions regarding quality assurance procedures and the congruence in demonstrations that supervisors must maintain. The participants’ frustration with the clinical supervisors’ differing views on clinical procedure techniques was evident. Insight into clinical teaching, learning and assessment revealed inconsistencies among clinical supervisors despite the existence of a procedure guide. Using the same procedure and marking guideline to teach and assess undergraduate nursing students could ensure the congruence of clinical teaching even though the intricacies of the procedures may vary. Having congruent clinical procedures helps students to adjust to clinical practice (Thurling et al. 2017). The subsequent subtheme, which is concerned with how the clinical supervisor assesses a formative skill, was prompted by this.

#### Sub-theme 1.3: Formative assessment procedures

The formative assessment process is a planned procedure utilised by clinical supervisors and students who offer actionable feedback after an assessment to modify ongoing teaching and learning practices (Solheim et al. 2017). Formative assessment procedures occur within the teaching and learning process and aim to advance the development of students’ competencies (SAQA 2005). Minimal time, however, is being spend on formative assessment procedures, and this is being questioned by the participants:

‘… how can a supervisor do a procedure for you, an emergency procedure, emergency training procedure for five minutes and then you’re done.’ (Group 2, Participant 6, Focus group interview)

The emergency assessment procedures in particular appeared to be conducted speedily. The given comment suggests that some participants are being sceptical about the rather quick formative assessment procedures:

‘… for us we’re just happy that we’re getting marks and all that but it doesn’t help us at the end… she will give you hundred percent, okay that’s fine, but it doesn’t help you.’ (Group 2, Participant 6, Focus group interview)

Participants expressed their satisfaction with their inflated grades for these rather quick procedures. However, they were unsure if they were adequately trained to successfully complete the final exam. Additionally, they should be well prepared to provide essential healthcare care, particularly in the area of emergency care, to communities where they are the only healthcare provider (Bell & Brysiewicz 2020). Insight into how the clinical supervisor assesses a formative procedure gives more understanding of how they should advance the development of students’ competencies. Assessments are essential and help with student development and training (Bearman et al. 2018). Actionable feedback after a planned formative assessment is important for development. Clinical supervisors need to ensure that they provide quality feedback to advance the development of student competence.

The realities of clinical supervision regarding the clinical teaching, learning and assessment revealed pertinent concerns of the undergraduate nursing students. Emphasising the importance of clinical teaching, learning and assessment will encourage clinical competence while providing adequate guidance and fostering a supportive learning environment (Tuomikoski et al. 2020). It was quite interesting to learn from the interviews how much more openly the senior nursing participants spoke about clinical supervision concerns than the junior nursing participants. In addition, those who were less experienced benefited from the extra knowledge provided by the more experienced participants.

## Limitations

The study only included one undergraduate nursing institution. The clinical supervisors’ experiences were not investigated.

## Recommendations

It is important to ensure that clinical supervisors have the necessary expertise to guide and train undergraduate nursing students. A quality assurance procedure could be used to evaluate how to improve the clinical supervisor’s competence and function. Participation in in-service training and ongoing professional development in the form of educational clinical workshops could aid to improve their clinical assessment skills and expertise. To further understand how clinical supervision affects student pass rates, it is recommended that research be done in this area.

## Conclusion

The study explored undergraduate nursing students’ experiences with clinical supervision, and the emerging theme reflected the degree of responses and significance within the data set. The realities of supervision regarding assessment of undergraduate nursing students gave insight into the clinical assessments of the participants, which are conducted by the clinical supervisors. Clinical supervision plays a major role in the development and training of undergraduate nursing students. Exploring undergraduate nursing students’ experiences with clinical supervision uncovered their concerns about clinical assessments. The clinical supervisors’ function in terms of teaching, learning and assessment was acknowledged and requires continuous development. Spending time with the students while providing effective clinical teaching, developmental training and assessment can enable the students to become competent and self-assured professional nurses.
